# The genome sequence of a conopid fly, *Myopa testacea *(Linnaeus, 1767)

**DOI:** 10.12688/wellcomeopenres.20647.1

**Published:** 2024-02-29

**Authors:** Steven Falk, Oliver Poole, University of Oxford and Wytham Woods Genome Acquisition Lab, Darwin Tree of Life Barcoding collective, Wellcome Sanger Institute Tree of Life Management, Samples and Laboratory team, Wellcome Sanger Institute Scientific Operations: Sequencing Operations, Wellcome Sanger Institute Tree of Life Core Informatics team, Tree of Life Core Informatics collective, Darwin Tree of Life Consortium

**Affiliations:** 1 Independent researcher, Kenilworth, England, UK; 2 Ecology and Conservation, University of Exeter, Penryn, England, UK

**Keywords:** Myopa testacea, thick-headed fly, genome sequence, chromosomal, Diptera

## Abstract

We present a genome assembly from an individual male *Myopa testacea* (conopid fly; Arthropoda; Insecta; Diptera; Conopidae). The genome sequence is 243.3 megabases in span. Most of the assembly is scaffolded into 5 chromosomal pseudomolecules, including the X and Y sex chromosomes. The mitochondrial genome has also been assembled and is 17.61 kilobases in length. Gene annotation of this assembly on Ensembl identified 25,472 protein coding genes.

## Species taxonomy

Eukaryota; Metazoa; Eumetazoa; Bilateria; Protostomia; Ecdysozoa; Panarthropoda; Arthropoda; Mandibulata; Pancrustacea; Hexapoda; Insecta; Dicondylia; Pterygota; Neoptera; Endopterygota; Diptera; Brachycera; Muscomorpha; Eremoneura; Cyclorrhapha; Schizophora; Acalyptratae; Conopoidea; Conopidae; Myopinae; *Myopa*; *Myopa testacea* (Linnaeus, 1767) (NCBI:txid2867102).

## Background

*Myopa testacea* (Diptera: Conopidae, Myopinae) is a common Palaearctic fly from the ‘bee-grabber’ family. This family name is given by the endoparasitic nature of its members, which are thought to prey on other insects, particularly aculeate Hymenoptera ( Stuke, 2017). The distinguishing features of *Myopa* species are their reddish-brown colour, large white inflated faces, and curling abdomens used by females to deliver their eggs to unsuspecting prey ( Smith, 1969). Although *M. testacea* is typically 7–10 mm long, they vary greatly in size, with individuals ranging from 4–11 mm ( Stuke & Clements, 2008).

*Myopa testacea* is univoltine and has a spring flight period coinciding with the emergence of mating solitary bees. This flight period can be extended to July or later where higher altitudes and northerly latitudes delay prey emergence ( Stuke & Clements, 2008). The larval development of this species has been recorded to occur in the adult mining bees, *Andrena scotica* and *Andrena vaga*, however further research into their biology is required to understand the true extent and flexibility of host choice ( Stuke & Clements, 2008). Being the most common *Myopa* species in the UK, the role of *M. testacea* as pollinator could be significant since it can be found feeding on the same catkins and flowers that attract the solitary bees which it parasitises. Recent application of DNA barcoding in *Myopa* host-parasite interactions has successfully identified a fourth host species in this genus ( Smit *et al.*, 2018). Here we add to the growing database of chromosomal DNA for *Myopa testacea* in the hope that it will be used to help untangle the complex taxonomy in this genus and aid further investigation into the biology and ecology of this species.

## Genome sequence report

**Figure 1.**
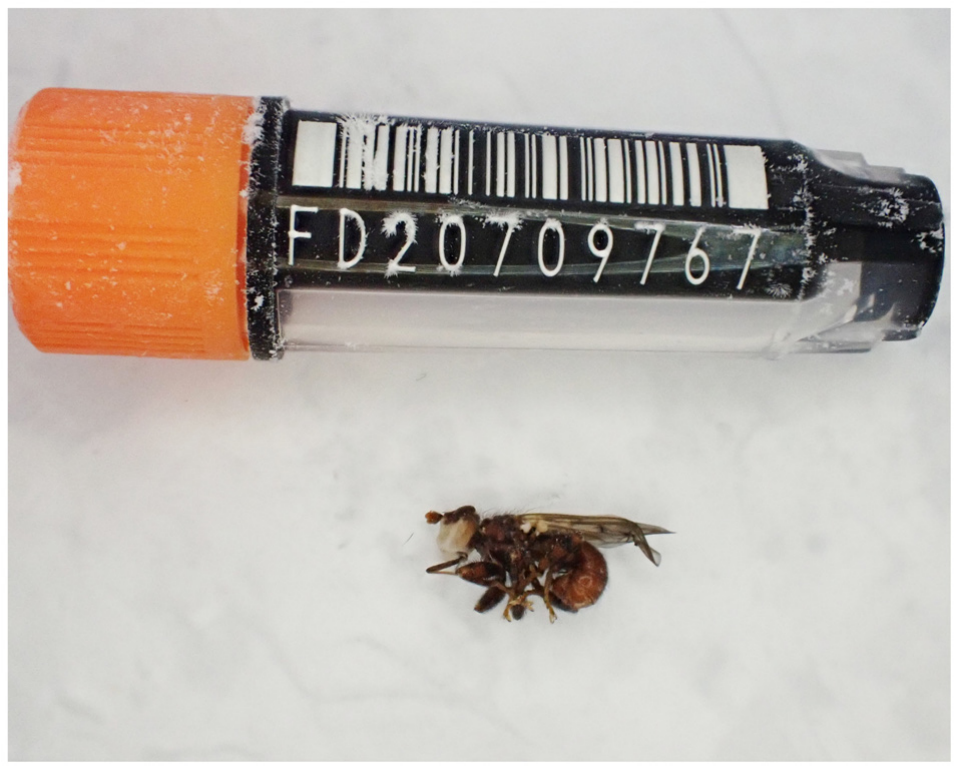
Photograph of the *Myopa testacea* (idMyoTest1) specimen used for genome sequencing.

The genome was sequenced from one male *Myopa testacea* ( Figure 1) collected from Wytham Woods, Oxfordshire, UK (51.76, –1.34). A total of 117-fold coverage in Pacific Biosciences single-molecule HiFi long reads was generated. Primary assembly contigs were scaffolded with chromosome conformation Hi-C data. Manual assembly curation corrected 201 missing joins or mis-joins and removed 28 haplotypic duplications, reducing the assembly length by 0.71% and the scaffold number by 76.15%, and increasing the scaffold N50 by 3.64%.

**Figure 2.**
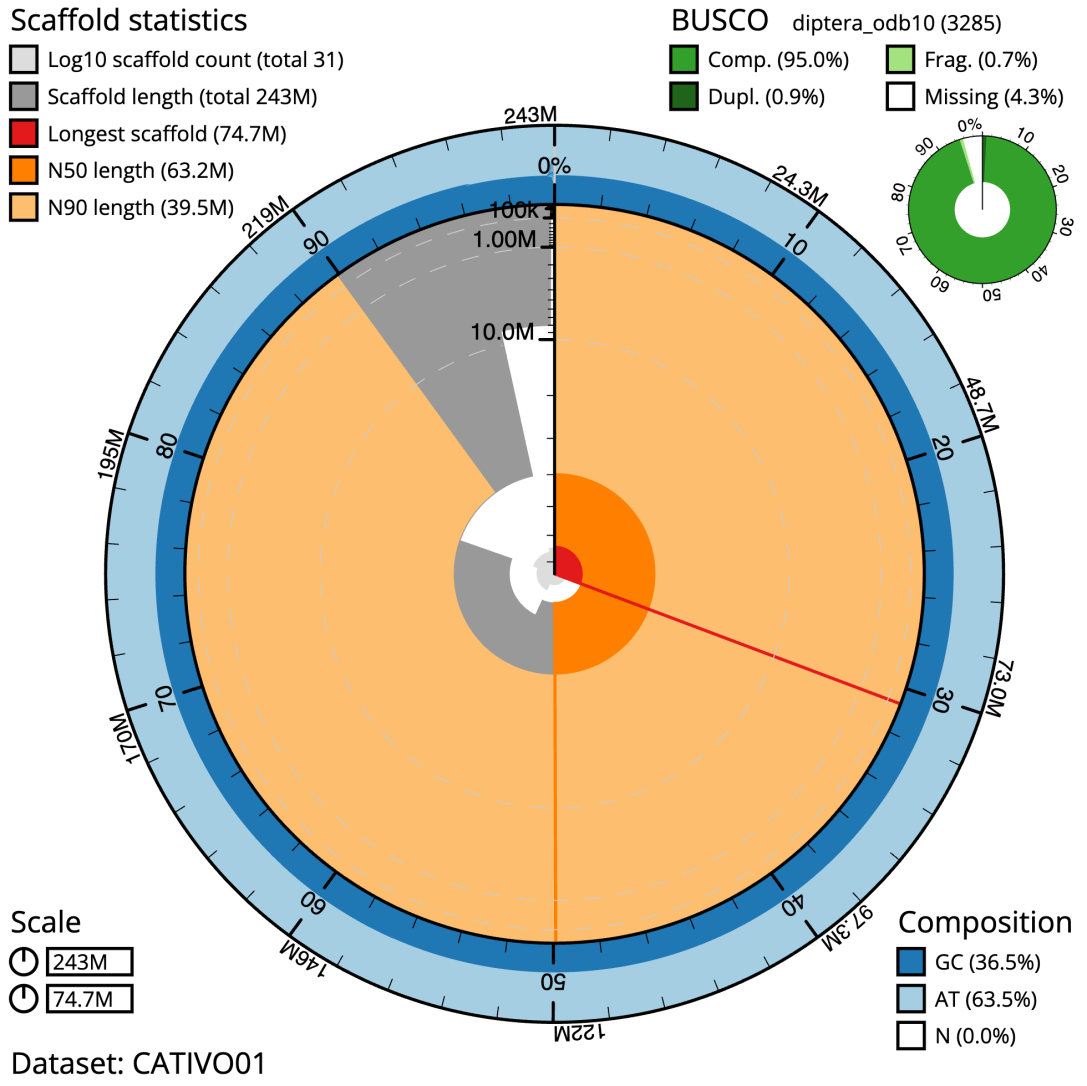
Genome assembly of *Myopa testacea*, idMyoTest1.1: metrics. The BlobToolKit Snailplot shows N50 metrics and BUSCO gene completeness. The main plot is divided into 1,000 size-ordered bins around the circumference with each bin representing 0.1% of the 243,347,938 bp assembly. The distribution of scaffold lengths is shown in dark grey with the plot radius scaled to the longest scaffold present in the assembly (74,748,633 bp, shown in red). Orange and pale-orange arcs show the N50 and N90 scaffold lengths (63,215,029 and 39,529,147 bp), respectively. The pale grey spiral shows the cumulative scaffold count on a log scale with white scale lines showing successive orders of magnitude. The blue and pale-blue area around the outside of the plot shows the distribution of GC, AT and N percentages in the same bins as the inner plot. A summary of complete, fragmented, duplicated and missing BUSCO genes in the diptera_odb10 set is shown in the top right. An interactive version of this figure is available at https://blobtoolkit.genomehubs.org/view/Myopa%20testacea/dataset/CATIVO01/snail.

**Figure 3.**
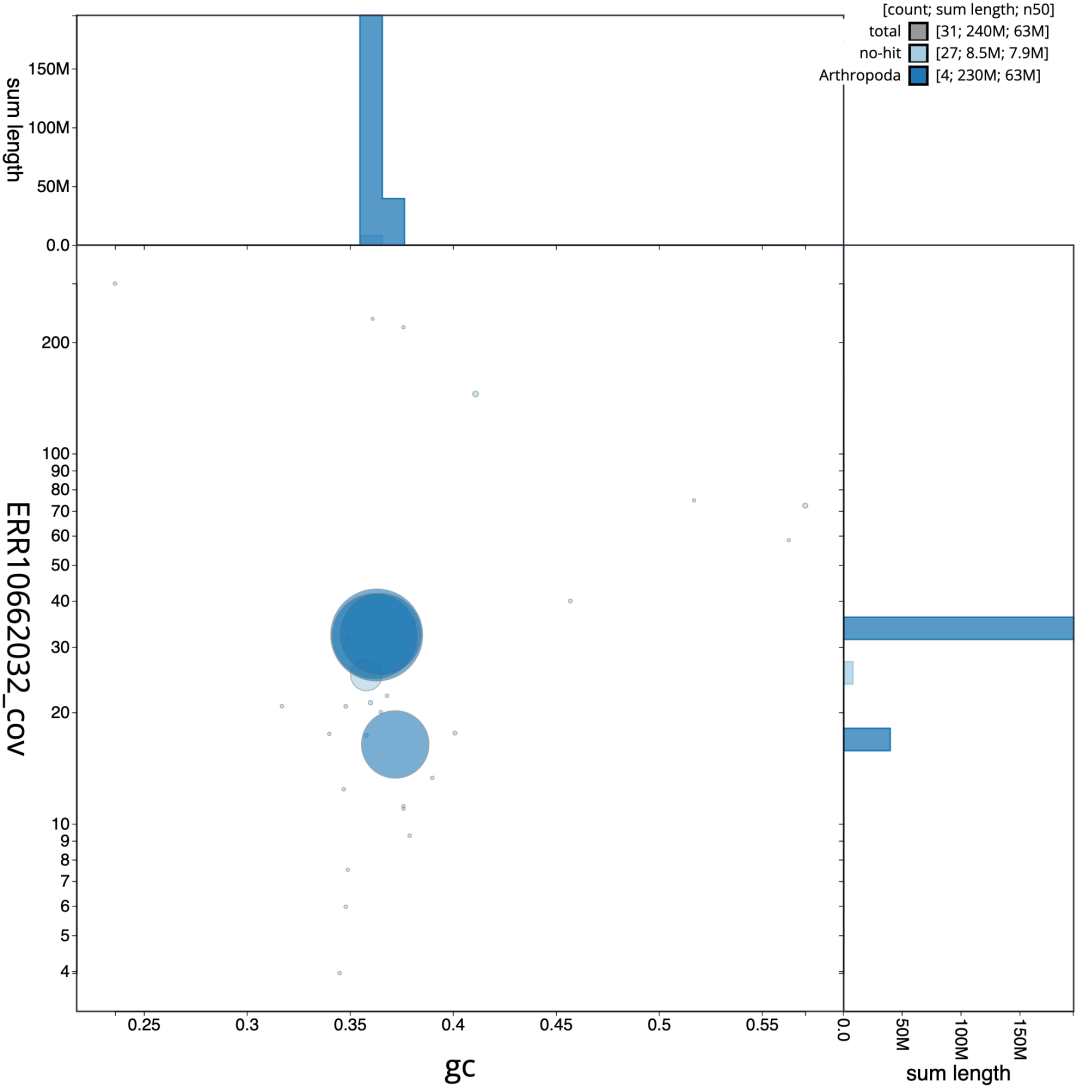
Genome assembly of *Myopa testacea*, idMyoTest1.1: BlobToolKit GC-coverage plot. Scaffolds are coloured by phylum. Circles are sized in proportion to scaffold length. Histograms show the distribution of scaffold length sum along each axis. An interactive version of this figure is available at https://blobtoolkit.genomehubs.org/view/Myopa%20testacea/dataset/CATIVO01/blob.

**Figure 4.**
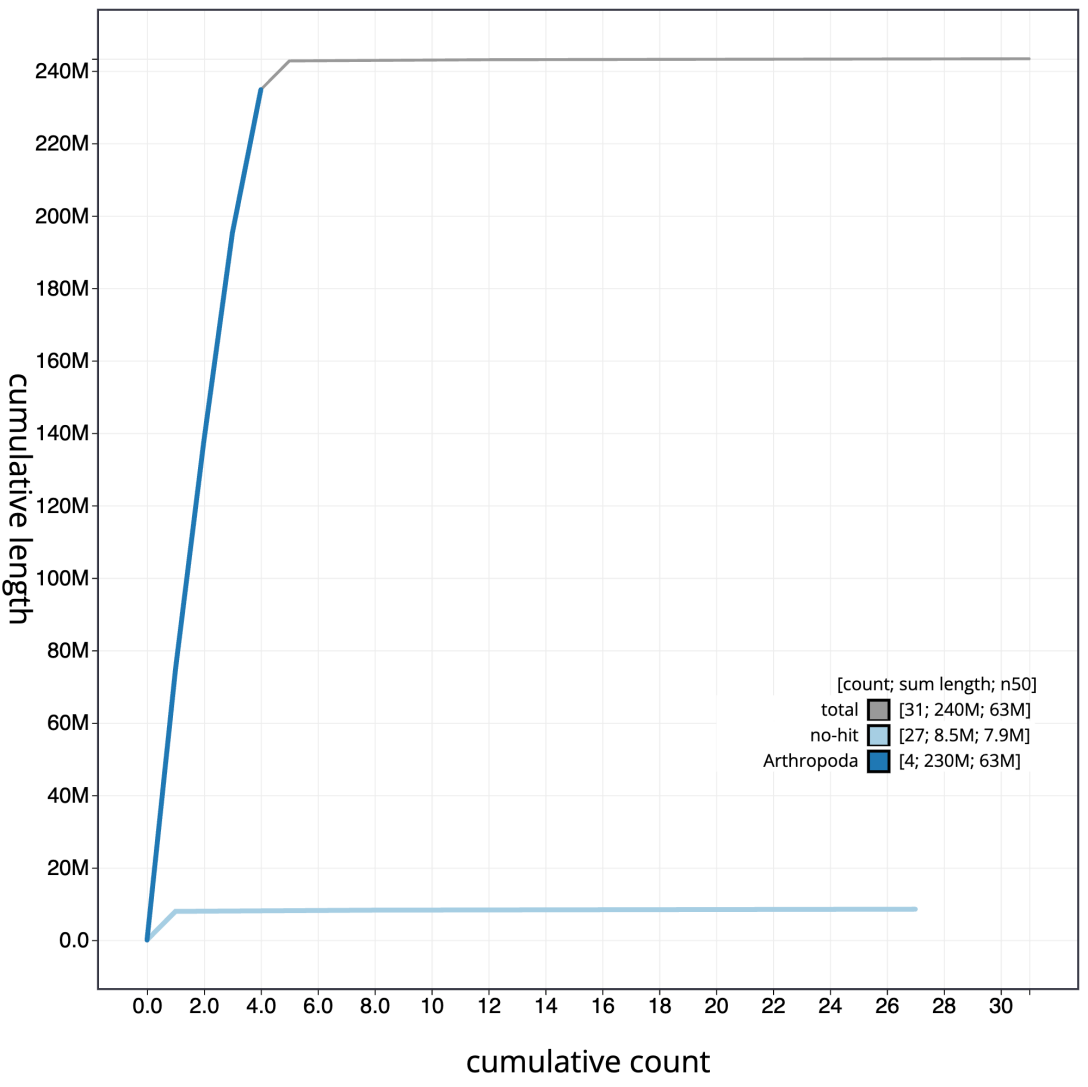
Genome assembly of *Myopa testacea*, idMyoTest1.1: BlobToolKit cumulative sequence plot. The grey line shows cumulative length for all scaffolds. Coloured lines show cumulative lengths of scaffolds assigned to each phylum using the buscogenes taxrule. An interactive version of this figure is available at https://blobtoolkit.genomehubs.org/view/Myopa%20testacea/dataset/CATIVO01/cumulative.

**Figure 5.**
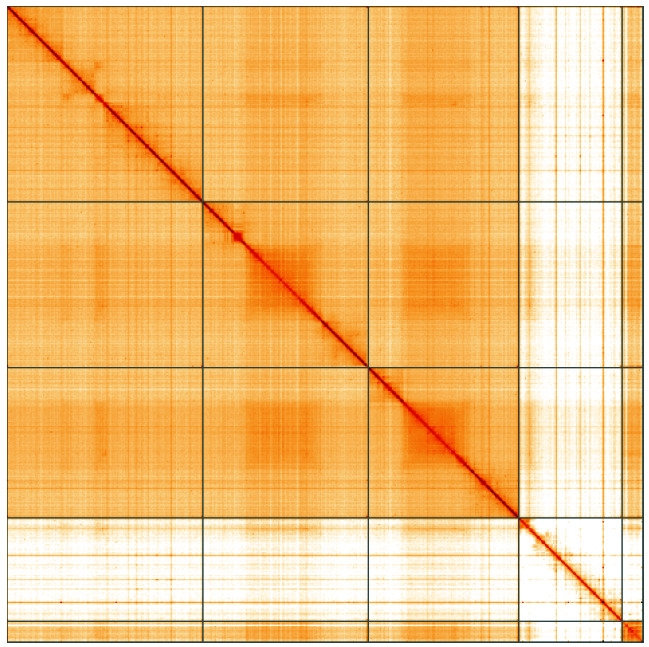
Genome assembly of *Myopa testacea*, idMyoTest1.1: Hi-C contact map of the idMyoTest1.1 assembly, visualised using HiGlass. Chromosomes are shown in order of size from left to right and top to bottom. An interactive version of this figure may be viewed at https://genome-note-higlass.tol.sanger.ac.uk/l/?d=Rylu9Mn1RYGQo-JpaIBmig.

The final assembly has a total length of 243.3 Mb in 30 sequence scaffolds with a scaffold N50 of 63.2 Mb ( Table 1). The snailplot in Figure 2 provides a summary of the assembly statistics, while the distribution of assembly scaffolds on GC proportion and coverage is shown in Figure 3. The cumulative assembly plot in Figure 4 shows curves for subsets of scaffolds assigned to different phyla. Most (99.78%) of the assembly sequence was assigned to 5 chromosomal-level scaffolds, representing 3 autosomes and the X and Y sex chromosomes. The sex chromosomes were identified by read depth. Chromosome-scale scaffolds confirmed by the Hi-C data are named in order of size ( Figure 5; Table 2). While not fully phased, the assembly deposited is of one haplotype. Contigs corresponding to the second haplotype have also been deposited. The mitochondrial genome was also assembled and can be found as a contig within the multifasta file of the genome submission.

**Table 1.** Genome data for *Myopa testacea*, idMyoTest1.1.

Project accession data		
Assembly identifier	idMyoTest1.1	
Species	*Myopa testacea*	
Specimen	idMyoTest1	
NCBI taxonomy ID	2867102	
BioProject	PRJEB58147	
BioSample ID	SAMEA10166770	
Isolate information	idMyoTest1, male: whole organism (DNA and Hi-C)	
Assembly metrics *		*Benchmark*
Consensus quality (QV)	58.8	*≥ 50*
*k*-mer completeness	100%	*≥ 95%*
BUSCO **	C:95.0%[S:94.2%,D:0.9%], F:0.7%,M:4.3%,n:3,285	*C ≥ 95%*
Percentage of assembly mapped to chromosomes	99.78%	*≥ 95%*
Sex chromosomes	X and Y	*localised homologous pairs*
Organelles	Mitochondrial genome assembled	*complete single alleles*
Raw data accessions		
PacificBiosciences SEQUEL II	ERR10662032	
Hi-C Illumina	ERR10659261	
Genome assembly		
Assembly accession	GCA_949629155.1	
*Accession of alternate haplotype*	GCA_949629165.2	
Span (Mb)	243.3	
Number of contigs	623	
Contig N50 length (Mb)	0.7	
Number of scaffolds	30	
Scaffold N50 length (Mb)	63.2	
Longest scaffold (Mb)	74.8	
Genome annotation		
Number of protein-coding genes	25,472	
Number of gene transcripts	26,236	

* Assembly metric benchmarks are adapted from column VGP-2020 of “Table 1: Proposed standards and metrics for defining genome assembly quality” from ( Rhie *et al.*, 2021). ** BUSCO scores based on the diptera_odb10 BUSCO set using v5.3.2. C = complete [S = single copy, D = duplicated], F = fragmented, M = missing, n = number of orthologues in comparison. A full set of BUSCO scores is available at https://blobtoolkit.genomehubs.org/view/Myopa testacea/dataset/CATIVO01/busco.

**Table 2.** Chromosomal pseudomolecules in the genome assembly of *Myopa testacea*, idMyoTest1.

INSDC accession	Chromosome	Length (Mb)	GC%
OX451345.1	1	74.75	36.5
OX451346.1	2	63.22	36.0
OX451347.1	3	57.32	36.5
OX451348.1	X	39.53	37.0
OX451349.1	Y	7.93	36.0
OX451350.1	MT	0.02	23.5

The estimated Quality Value (QV) of the final assembly is 58.8 with *k*-mer completeness of 100%, and the assembly has a BUSCO v5.3.2 completeness of 95.0% (single = 94.2%, duplicated = 0.9%), using the diptera_odb10 reference set ( *n* = 3,285).

Metadata for specimens, barcode results, spectra estimates, sequencing runs, contaminants and pre-curation assembly statistics are given at https://links.tol.sanger.ac.uk/species/2867102.

## Genome annotation report

The *Myopa testacea* genome assembly (GCA_949629155.1) was annotated using the Ensembl rapid annotation pipeline ( Table 1; https://rapid.ensembl.org/Myopa_testacea_GCA_949629155.1/Info/Index). The resulting annotation includes 25,472 transcribed mRNAs from 26,236 protein-coding genes.

## Methods

### Sample acquisition and nucleic acid extraction

A male *Myopa testacea* (specimen ID Ox001290, ToLID idMyoTest1) was netted in Wytham Woods, Oxfordshire, UK (latitude 51.76, longitude –1.34) on 2021-04-23. The specimen was collected and identified by Steven Falk (independent researcher) and snap-frozen on dry ice.

Protocols developed by the Wellcome Sanger Institute (WSI) Tree of Life Core Laboratory have been deposited on protocols.io ( Denton *et al.*, 2023b). The workflow for high molecular weight (HMW) DNA extraction at the WSI includes a sequence of procedures: sample preparation; sample homogenisation, DNA extraction, fragmentation, and clean-up. The idMyoTest1 sample was weighed and dissected on dry ice ( Jay *et al.*, 2023), with tissue set aside for Hi-C sequencing. Tissue from the thorax was homogenised using a PowerMasher II tissue disruptor ( Denton *et al.*, 2023a). HMW DNA was extracted in the WSI Scientific Operations core using the Automated MagAttract v2 protocol ( Oatley *et al.*, 2023). HMW DNA was sheared into an average fragment size of 12–20 kb in a Megaruptor 3 system with speed setting 31 ( Bates *et al.*, 2023). Sheared DNA was purified by solid-phase reversible immobilisation ( Strickland *et al.*, 2023): in brief, the method employs a 1.8X ratio of AMPure PB beads to sample to eliminate shorter fragments and concentrate the DNA. The concentration of the sheared and purified DNA was assessed using a Nanodrop spectrophotometer and Qubit Fluorometer and Qubit dsDNA High Sensitivity Assay kit. Fragment size distribution was evaluated by running the sample on the FemtoPulse system.

### Sequencing

Pacific Biosciences HiFi circular consensus DNA sequencing libraries were constructed according to the manufacturers’ instructions. DNA sequencing was performed by the Scientific Operations core at the WSI on a Pacific Biosciences SEQUEL II instrument. Hi-C data were also generated from remaining tissue of idMyoTest1 using the Arima2 kit and sequenced on the Illumina NovaSeq 6000 instrument.

### Genome assembly, curation and evaluation

Assembly was carried out with Hifiasm ( Cheng *et al.*, 2021) and haplotypic duplication was identified and removed with purge_dups ( Guan *et al.*, 2020). The assembly was then scaffolded with Hi-C data ( Rao *et al.*, 2014) using YaHS ( Zhou *et al.*, 2023). The assembly was checked for contamination and corrected as described previously ( Howe *et al.*, 2021). Manual curation was performed using HiGlass ( Kerpedjiev *et al.*, 2018) and Pretext ( Harry, 2022). The mitochondrial genome was assembled using MitoHiFi ( Uliano-Silva *et al.*, 2023), which runs MitoFinder ( Allio *et al.*, 2020) or MITOS ( Bernt *et al.*, 2013) and uses these annotations to select the final mitochondrial contig and to ensure the general quality of the sequence.

A Hi-C map for the final assembly was produced using bwa-mem2 ( Vasimuddin *et al.*, 2019) in the Cooler file format ( Abdennur & Mirny, 2020). To assess the assembly metrics, the *k*-mer completeness and QV consensus quality values were calculated in Merqury ( Rhie *et al.*, 2020). This work was done using Nextflow ( Di Tommaso *et al.*, 2017) DSL2 pipelines “sanger-tol/readmapping” ( Surana *et al.*, 2023a) and “sanger-tol/genomenote” ( Surana *et al.*, 2023b). The genome was analysed within the BlobToolKit environment ( Challis *et al.*, 2020) and BUSCO scores ( Manni *et al.*, 2021; Simão *et al.*, 2015) were calculated.

Table 3 contains a list of relevant software tool versions and sources.

**Table 3.** Software tools: versions and sources.

Software tool	Version	Source
BlobToolKit	4.2.1	https://github.com/blobtoolkit/blobtoolkit
BUSCO	5.3.2	https://gitlab.com/ezlab/busco
Hifiasm	0.16.1-r375	https://github.com/chhylp123/hifiasm
HiGlass	1.11.6	https://github.com/higlass/higlass
Merqury	MerquryFK	https://github.com/thegenemyers/MERQURY.FK
MitoHiFi	2	https://github.com/marcelauliano/MitoHiFi
PretextView	0.2	https://github.com/wtsi-hpag/PretextView
purge_dups	1.2.3	https://github.com/dfguan/purge_dups
sanger-tol/ genomenote	v1.0	https://github.com/sanger-tol/genomenote
sanger-tol/ readmapping	1.1.0	https://github.com/sanger-tol/readmapping/tree/1.1.0
YaHS	1.1a.2	https://github.com/c-zhou/yahs

### Genome annotation

The BRAKER2 pipeline ( Brůna *et al.*, 2021) was used in the default protein mode to generate annotation for the *Myopa testacea* assembly (GCA_949629155.1) in Ensembl Rapid Release.

### Wellcome Sanger Institute – Legal and Governance

The materials that have contributed to this genome note have been supplied by a Darwin Tree of Life Partner. The submission of materials by a Darwin Tree of Life Partner is subject to the ‘Darwin Tree of Life Project Sampling Code of Practice’, which can be found in full on the Darwin Tree of Life website here. By agreeing with and signing up to the Sampling Code of Practice, the Darwin Tree of Life Partner agrees they will meet the legal and ethical requirements and standards set out within this document in respect of all samples acquired for, and supplied to, the Darwin Tree of Life Project. 

Further, the Wellcome Sanger Institute employs a process whereby due diligence is carried out proportionate to the nature of the materials themselves, and the circumstances under which they have been/are to be collected and provided for use. The purpose of this is to address and mitigate any potential legal and/or ethical implications of receipt and use of the materials as part of the research project, and to ensure that in doing so we align with best practice wherever possible. The overarching areas of consideration are:

•     Ethical review of provenance and sourcing of the material

•     Legality of collection, transfer and use (national and international) 

Each transfer of samples is further undertaken according to a Research Collaboration Agreement or Material Transfer Agreement entered into by the Darwin Tree of Life Partner, Genome Research Limited (operating as the Wellcome Sanger Institute), and in some circumstances other Darwin Tree of Life collaborators.

## Data Availability

European Nucleotide Archive: *Myopa testacea*. Accession number PRJEB58147; https://identifiers.org/ena.embl/PRJEB58147 ( Wellcome Sanger Institute, 2023). The genome sequence is released openly for reuse. The *Myopa testacea* genome sequencing initiative is part of the Darwin Tree of Life (DToL) project. All raw sequence data and the assembly have been deposited in INSDC databases. Raw data and assembly accession identifiers are reported in Table 1.

## Author information

Members of the University of Oxford and Wytham Woods Genome Acquisition Lab are listed here: https://doi.org/10.5281/zenodo.7125292.

Members of the Darwin Tree of Life Barcoding collective are listed here: https://doi.org/10.5281/zenodo.4893703.

Members of the Wellcome Sanger Institute Tree of Life programme are listed here: https://doi.org/10.5281/zenodo.4783585.

Members of Wellcome Sanger Institute Scientific Operations: Sequencing Operations collective are listed here: https://doi.org/10.5281/zenodo.10043364.

Members of the Tree of Life Core Informatics collective are listed here: https://doi.org/10.5281/zenodo.5013541.

Members of the Darwin Tree of Life Consortium are listed here: https://doi.org/10.5281/zenodo.4783558.
