# Supplementary material for: The genome sequence of a conopid fly, Myopa testacea (Linnaeus, 1767)
Source: Wellcome Open Res. 2024 Feb 29;9:99. [Version 1] doi: 10.12688/wellcomeopenres.20647.1 (PMC11058451; doi:10.12688/wellcomeopenres.20647.1)
Supplement: Peer review history

# Reviewer report: Reviewer response for version 1

**DOI:** 10.21956/wellcomeopenres.22851.r79063

**Published:** 2024-05-13

**Authors:** Ming-Der Lin

**Affiliations:** 1 Tzu Chi University, Hualien, Taiwan

This study presents the chromosomal-level genome assembly of a male *Myopa testacea* (conopid fly), a common Palaearctic endoparasitic fly known for preying on aculeate Hymenoptera. The assembly spans 243.3 megabases, scaffolded into five chromosomal pseudomolecules, and includes a 17.61 kilobase mitochondrial genome. This assembly identifies 25,472 protein-coding genes through the BRAKER2 pipeline. The manuscript is well-structured as a genome report. However, there are some issues that need to be addressed to enhance the completeness of the manuscript:

1. The authors indicate that “Manual assembly curation corrected missing joins or mis-joins and removed 28 haplotypic duplications.” Should the authors describe how the missing joins and haplotypic duplications were identified?
2. The authors used the BRAKER2 pipeline to annotate the *Myopa testacea* genome. Could the authors provide BUSCO values for the annotation results to demonstrate the completeness of the annotation?
3. Did the authors use programs such as the NCBI Foreign Contamination Screen (FCS) to remove possible contamination sequences?

Are sufficient details of methods and materials provided to allow replication by others?

Partly

Is the rationale for creating the dataset(s) clearly described?

Yes

Are the datasets clearly presented in a useable and accessible format?

Yes

Are the protocols appropriate and is the work technically sound?

Yes

Reviewer Expertise:

Genomics and developmental biology

I confirm that I have read this submission and believe that I have an appropriate level of expertise to confirm that it is of an acceptable scientific standard.

# Reviewer report: Reviewer response for version 1

**DOI:** 10.21956/wellcomeopenres.22851.r79067

**Published:** 2024-04-29

**Authors:** Xiaodong Cai

**Affiliations:** 1 China Agricultural University, Beijing, China

This article reports the assembly of the chromosome-level genome of *Myopa testacea* through sequencing of a male specimen, with a genome size of 243.3 Mb and a Contig N50 of 0.7 Mb. Additionally, 25472 protein-coding genes were annotated. In addition to the genome, the mitochondrial genome of *Myopa testacea* was also assembled. All data presented in this study have been released and are available for public access, and detailed methods have been provided. The data provided in this study serve as important reference data for future genomic and phylogenetic studies of Diptera. However, I still have some comments on this article.

**Abstract**

 The completeness of the mitochondrial genome, whether it forms a complete circle, is a crucial aspect that should be clarified.

**Figure 1**

 The quality of the insect images is poor, making it difficult to discern their primary morphological features. It is recommended to provide high-quality specimen photographs.

**Genome sequence report**

 1. The chromosome genome obtained in the article suggests conducting a synteny analysis with the model organism Drosophila, to better demonstrate to readers the size and structural features of the chromosomes.

 2. The manuscript mentions the assembly of the mitochondrial genome, yet lacks elaboration on its size, structure, and whether it forms a complete circle. It is suggested to provide a brief overview of these aspects.

**Genome annotation report**

 The article annotates 26,236 protein-coding genes in the genome. It is well known that repetitive sequences and non-coding RNAs play important roles in organisms. However, the annotation status of repetitive sequences and non-coding RNAs is not mentioned in the text. If possible, it is recommended to add this information to provide readers with a more comprehensive understanding of the genome.

Are sufficient details of methods and materials provided to allow replication by others?

Yes

Is the rationale for creating the dataset(s) clearly described?

Yes

Are the datasets clearly presented in a useable and accessible format?

Yes

Are the protocols appropriate and is the work technically sound?

Yes

Reviewer Expertise:

Systematic entomology

I confirm that I have read this submission and believe that I have an appropriate level of expertise to confirm that it is of an acceptable scientific standard.

# Reviewer report: Reviewer response for version 1

**DOI:** 10.21956/wellcomeopenres.22851.r79061

**Published:** 2024-04-17

**Authors:** Ravikumar D Dodiya

**Affiliations:** 1 Sardarkrushinagar Dantiwada Agricultural University, Sardarkrushinagar, Dantiwada, India

Summary:

 The article presents the genome assembly of *Myopa testacea*, a species of conopid fly. The genome sequence spans 243.3 megabases and includes chromosomal pseudomolecules, including the X and Y sex chromosomes. Additionally, the mitochondrial genome, 17.61 kilobases in length, has been assembled. Gene annotation identified 25,472 protein-coding genes using Ensembl.

*Myopa testacea* is described as a common Palaearctic fly from the Conopidae family known for its endoparasitic nature, particularly preying on other insects like solitary bees. The species exhibits distinctive features such as a reddish-brown color, large white faces, and curling abdomens in females for laying eggs on prey.

 The genome was sequenced from a male specimen collected in Wytham Woods, Oxfordshire, UK, with extensive coverage using Pacific Biosciences HiFi long reads and scaffolded with chromosome conformation Hi-C data. Manual curation corrects assembly errors and improves scaffold quality.

 Methods for sample acquisition, nucleic acid extraction, and sequencing are detailed, including the use of high molecular weight DNA extraction, Hi-C data generation, and quality assessment of the assembly.

 Overall, the genome assembly provides valuable genetic information for further research into the biology, ecology, and taxonomy of *Myopa testacea* and contributes to understanding the complex interactions within its ecosystem.

Are sufficient details of methods and materials provided to allow replication by others?

Yes

Is the rationale for creating the dataset(s) clearly described?

Yes

Are the datasets clearly presented in a useable and accessible format?

Yes

Are the protocols appropriate and is the work technically sound?

Yes

Reviewer Expertise:

Biological control, Molecular Research

I confirm that I have read this submission and believe that I have an appropriate level of expertise to confirm that it is of an acceptable scientific standard.
